# AI expectation violations and learner engagement in EFL contexts: a cognitive-affective recovery model

**DOI:** 10.3389/fpsyg.2026.1707116

**Published:** 2026-02-05

**Authors:** Jiawen Yu, Xianyan Dai, Baoyu Qiu, Wanling Guo, Rong Wang, Meng Na

**Affiliations:** 1 School of Foreign Languages, Nanfang College, Guangzhou, Guangdong, China; 2 Faculty of Education, Language, Psychology, and Music, SEGi University, Petaling Jaya, Selangor, Malaysia; 3 School of Chinese and International Education, Guangzhou International Economics College, Guangzhou, China; 4 Faculty of the Graduate School, Emilio Aguinaldo College, Manila, Philippines; 5 Faculty of Social Science, University of Macau, Taipa, China; 6 Foreign Language College, Hanshan Normal University, Chaozhou, China; 7 Linguistics and Applied Linguistics Center, Guangdong University of Foreign Studies, Guangzhou, Guangdong, China; 8 School of Educational Information Technology, South China Normal University, Guangzhou, Guangdong, China; 9 Graduate School of Business, Universiti Kebangsaan Malaysia, Bangi, Selangor, Malaysia

**Keywords:** AI adaptivity, cognitive reappraisal, digital grit, digital resilience, EFL education, learning engagement, trust recovery

## Abstract

Artificial Intelligence (AI) is increasingly deployed in English-as-a-foreign-language (EFL) education, offering adaptive feedback, automated evaluation, and personalized learning pathways. However, existing research overwhelmingly emphasizes AI adoption and performance benefits, while largely overlooking what happens when AI systems fail to meet learner expectations and how learners recover from such failures. As a result, the cognitive–affective processes through which expectation violations translate into disengagement—or are mitigated through recovery—remain under-theorized and empirically unexplored. Addressing this gap, this study proposes and tests a cognitive–affective recovery model of learner engagement in AI-supported EFL contexts. Drawing on Expectation Violation Theory (EVT), Cognitive Appraisal Theory (CAT), and Digital Divide/Resilience Theory, the model explains how expectation violations influence engagement and how cognitive reappraisal and trust recovery mediate this relationship, while digital grit conditions learners’ ability to persist following setbacks. A two-wave survey of 298 Chinese EFL learners from urban and rural settings, including both university students and private institute learners, was analyzed using Partial Least Squares Structural Equation Modeling (PLS-SEM). Results show that expectation violations significantly reduce learner engagement, but perceived AI adaptivity narrows the adaptation gap and activates recovery processes. Cognitive reappraisal and trust recovery emerged as key mediating mechanisms, while digital grit moderated critical pathways by sustaining engagement under adverse conditions. By shifting the focus from AI success narratives to failure-and-recovery dynamics, this study advances theory on AI–learner interaction and offers practical guidance for designing resilient, trust-sensitive, and equity-oriented AI systems in language education.

## 1 Introduction

Artificial intelligence (AI) has rapidly become embedded in English-as-a-foreign-language (EFL) education, promising adaptive learning pathways, real-time feedback, and scalable instructional support. By 2024, approximately 62% of language-learning platforms worldwide had incorporated AI functionalities such as chatbots, adaptive feedback engines, and automated writing evaluators (HolonIQ, 2024). China exemplifies this acceleration: its EdTech market reached USD 70 billion in 2023, with AI-supported English learning among the fastest-growing segments (Statista, 2024). In EFL contexts, AI tools are frequently promoted as a solution to long-standing challenges of large class sizes, limited teacher feedback, and heterogeneous learner needs, and are therefore assumed to enhance learner engagement and achievement through personalization and immediacy (Xu and Wang, 2022; Wei, 2023).

However, the central challenge facing AI-supported EFL learning is no longer simply whether learners adopt AI technologies, but what happens after adoption when AI systems fail to meet learner expectations. Learners often enter AI-mediated learning environments with strong assumptions about accuracy, responsiveness, and contextual sensitivity. In practice, many encounter generic praise (“good job,” “try again”), misclassification of grammar and discourse features, or feedback that fails to account for proficiency level, task purpose, or rhetorical intent. Such expectation–experience mismatches are increasingly reported to generate frustration, confusion, mistrust, and disengagement (Fu et al., 2024; Wang and Guo, 2024). Empirical evidence suggests that disappointment can emerge quickly: although initial enthusiasm for AI tools is typically high, nearly half of EFL learners report dissatisfaction within a few weeks due to misaligned feedback and limited adaptivity (Yan et al., 2024). Even emotion-adaptive AI systems improve learning outcomes only when learners’ affective states are accurately detected, underscoring the fragility of engagement when AI performance deviates from expectations (Aliahmadi and Saravi-Moghadam, 2020; Aziz et al., 2025).

These failures are not abstract but highly visible in everyday EFL practice. Automated essay scorers may flag idiomatic expressions or hedging devices as errors; grammar checkers may overlook cohesion and discourse-level meaning; conversational agents may recycle template hints rather than offering scaffolded explanations. Such breakdowns are particularly discouraging when learners have been repeatedly told that “AI is more precise than humans,” amplifying the psychological impact of unmet expectations. In these situations, engagement is no longer driven by novelty or perceived usefulness, but by how learners interpret, emotionally respond to, and recover from disappointment.

Despite growing evidence of these challenges, much of the AI-in-education literature continues to emphasize positive outcomes—performance gains (Pavlenko et al., 2024), enhanced emotional engagement (Huang and Mizumoto, 2024; Ahmed and Aziz, 2025), and improved self-regulation (Wang et al., 2024)—while comparatively overlooking the failure-to-recovery process that determines whether learners persist or disengage when AI underperforms. Two critical gaps follow. First, there is a theoretical gap: existing models rarely explain how expectation violations in AI-enhanced EFL settings translate into cognitive–affective responses such as disappointment or frustration, nor how learners recover—or fail to recover—through mechanisms such as cognitive reappraisal and trust rebuilding (Crompton et al., 2024; Fathi et al., 2024). Recovery is often implicitly assumed rather than explicitly theorized and tested. Second, there is an equity and contextual gap: prevailing frameworks tend to overlook how contextual constraints (e.g., connectivity, device access, reliance on a single platform) and learner dispositions (e.g., perseverance in digital environments) condition whether engagement can be sustained following AI failures.

These gaps are especially consequential in China’s high-stakes EFL ecosystem. Examination pressure, strong performance orientation, and cultural norms that emphasize correctness and authority can heighten expectations for technological precision (Wei et al., 2022). In such contexts, AI feedback is easily interpreted as authoritative rather than provisional, making expectation violations psychologically costly and potentially demotivating. Cultural sensitivities surrounding error, face, and effort investment may further amplify disappointment when AI contradicts a learner’s perceived progress. At the same time, contextual inequality intensifies vulnerability. According to the China Internet Network Information Center (2023), nearly 30% of rural students lack stable internet access or personal digital devices. For these learners, AI tools may function as a primary tutor rather than a supplementary aid; when such tools fail, both the psychological cost (e.g., loss of confidence, learned helplessness) and opportunity cost (e.g., lost practice time) are disproportionately high.

This study addresses these challenges by reframing AI-supported EFL learning around failure and recovery rather than success alone. Conceptually, we integrate Expectation Violation Theory (EVT) to model the triggering mechanism—the magnitude of mismatch between expected and perceived AI performance—with Cognitive Appraisal Theory (CAT) to explain recovery processes through cognitive reappraisal and trust recovery. In addition, a digital divide and resilience perspective is incorporated to explain heterogeneity in learner responses, highlighting why some learners persist through AI failures while others disengage. This integrated framework allows engagement to be understood not as a static outcome of AI adoption, but as a dynamic process shaped by violation, appraisal, and recovery.

The model is grounded in common EFL use cases where AI failures are salient and consequential, including grammar correction tools, automated essay scoring systems, chatbots, and adaptive vocabulary tutors. For example, when an automated writing evaluator systematically penalizes rhetorical moves typical of advanced EFL writing (e.g., hedging, metadiscourse), learners may either reinterpret the failure (“the model does not yet capture my argument”) and persist, or experience trust erosion and disengage. Understanding these divergent responses requires explicit attention to both cognitive and affective recovery mechanisms.

Accordingly, this study pursues four objectives. First, it examines how expectation violation magnitude relates to learner engagement in AI-enhanced EFL environments, explicitly focusing on when AI undermines rather than enhances engagement. Second, it tests whether cognitive reappraisal and trust recovery mediate this relationship, thereby specifying how learners recover from disappointment. Third, it investigates whether perceived AI adaptivity reduces expectation violations and facilitates recovery by narrowing the adaptation gap and signaling responsiveness. Fourth, it assesses the moderating role of digital grit and socio-digital context in shaping these pathways.

By centering disappointment, resilience, and context, this study makes three contributions. Theoretically, it advances a cognitive–affective recovery model that conceptualizes AI–learner interaction as a cycle of expectation violation and recovery, complementing dominant adoption- and performance-focused narratives. Methodologically, it operationalizes under-examined constructs—expectation violation magnitude, cognitive reappraisal, trust recovery, perceived AI adaptivity, and digital grit—using a time-lagged survey design capable of testing mediated and moderated effects. Practically, it offers guidance for designing resilient and trust-sensitive AI systems (e.g., adaptive scaffolding, transparency cues, calibration feedback) and for developing equity-oriented policies that mitigate digital disadvantage. Ultimately, this study seeks to explain not only whether AI supports EFL learning, but when, how, and for whom it does so.

## 2 Literature review

### 2.1 AI, expectation violation, and engagement in education

Research on artificial intelligence (AI) in education has expanded rapidly, with most studies emphasizing adoption, acceptance, and performance outcomes. Across EFL and broader educational contexts, AI-supported systems are widely reported to enhance personalization, efficiency, and learner engagement (Triberti et al., 2024; Al Nabhani et al., 2025). Parallel research in language education also shows that instructional innovations—such as project-based learning and digitally enriched pedagogies—can foster engagement, emotional involvement, and learning achievement when learners perceive instructional activities as meaningful and supportive (John and Levshits, 2024; Wang et al., 2023). Collectively, these studies tend to assume a linear success pathway, in which improved instructional design or AI functionality directly translates into positive engagement and achievement.

However, this success-oriented framing obscures a growing body of evidence showing that AI systems frequently fail to meet learner expectations in practice, particularly when pedagogical depth, contextual sensitivity, or emotional responsiveness are limited (Chichekian and Benteux, 2022). Expectation-related research demonstrates that expectation confirmation, rather than AI use per se, is a critical determinant of satisfaction, trust, and engagement (Dang et al., 2025). When AI-generated feedback is perceived as inaccurate, generic, or misaligned with learning goals, learners report frustration, reduced motivation, and diminished trust—not only toward the AI system but toward the instructional environment in which it is embedded (Frederick et al., 2025).

Importantly, these reactions are not merely technical responses to performance errors. They reflect interpretive judgments about AI competence, authority, and pedagogical legitimacy (Lee, 2024). In high-stakes learning contexts such as EFL, where learners often treat feedback as evaluative and consequential, unmet expectations can carry substantial emotional and motivational costs. Prior work in EFL education shows that emotions such as anxiety, disappointment, and frustration strongly shape achievement through engagement pathways (Wang et al., 2023), suggesting that AI-related expectation violations may have downstream effects well beyond immediate dissatisfaction.

Existing research further indicates that learner positioning matters. When learners are treated as passive recipients of AI output, expectation violations tend to be more damaging; conversely, opportunities for interaction, agency, or co-construction can buffer negative effects (Narang and Ahlbom, 2025). This insight resonates with findings from project-based and technology-enhanced learning research, where engagement is sustained not by tools alone, but by learners’ capacity to actively interpret, negotiate, and respond to instructional challenges (Dijo et al., 2024; John and Levshits, 2024). Yet, despite these parallels, AI-in-education research rarely theorizes how learners cognitively and emotionally recover when AI systems underperform.

As a result, the literature remains fragmented. Studies on AI adoption emphasize expectancy confirmation but provide limited insight into what happens when expectations are violated. Engagement studies document disengagement following frustration but rarely explain why some learners persist while others withdraw. EFL-focused research often reports dissatisfaction with AI feedback quality, yet treats these findings descriptively rather than analytically. Even broader work on AI-enabled personalized learning and STEAM education highlights design potential while underexamining learner responses to system imperfection (John, 2025).

This gap is consequential. Expectation violations generate cognitive dissonance and emotional strain, which can undermine motivation, engagement, and persistence (Habib et al., 2025). In AI-mediated environments, such violations may also trigger concerns about depersonalization, surveillance, or erosion of pedagogical boundaries (Seo et al., 2021). While structured AI feedback and scaffolding can support adaptation, their effectiveness depends on learners’ ability to reappraise disappointment and rebuild trust—processes that remain under-theorized and under-tested, particularly in high-stakes EFL contexts where reliance on AI is increasing (Ali et al., 2024; Slade et al., 2024).

In short, existing research establishes that expectation violations matter, but it does not explain how learners recover from them, why recovery varies across individuals, or how contextual constraints shape these outcomes. Addressing this blind spot requires a theoretical framework that captures both the triggering of disappointment and the cognitive–affective recovery processes through which engagement is either restored or lost. This study responds to that need by advancing a recovery-centered model of learner engagement in AI-supported EFL education.

### 2.2 Theoretical foundations for a cognitive–affective recovery perspective

To explain how learners respond to AI failures in EFL contexts, this study integrates Expectation Violation Theory (EVT), Cognitive Appraisal Theory (CAT), and Digital Divide/Resilience Theory into a unified cognitive–affective recovery perspective. Each theory illuminates a distinct stage of the learner experience, and their integration enables engagement to be conceptualized as a dynamic process rather than a static outcome of AI adoption. EVT explains why engagement is disrupted when AI underperforms, CAT explains how learners cognitively and emotionally regulate this disruption, and digital divide and resilience perspectives explain why recovery capacity varies across learners and contexts.

From an EVT perspective, learners enter AI-supported EFL environments with expectations regarding accuracy, personalization, and instructional authority. When AI systems—such as automated writing evaluators, grammar correction tools, or conversational agents—produce generic feedback, misclassify linguistic features, or fail to respond to learner intent, these expectations are violated. The magnitude of this mismatch between anticipated and actual performance constitutes expectation violation magnitude, which triggers negative cognitive and emotional reactions. EVT predicts that stronger violations intensify dissatisfaction and undermine engagement, a pattern supported by evidence showing rapid abandonment of AI tools among EFL learners following repeated misalignments (Yan et al., 2024). However, EVT alone cannot explain why expectation violations do not uniformly result in disengagement.

Cognitive Appraisal Theory provides the missing explanatory link by accounting for how learners interpret and regulate emotionally significant events. CAT posits that outcomes are shaped not simply by the occurrence of a negative event, but by how individuals appraise its meaning and mobilize coping strategies. Applied to AI-supported EFL learning, expectation violations may prompt learners to engage in cognitive reappraisal—reinterpreting AI failure as temporary, system-limited, or non-diagnostic of personal ability. Through such reappraisal, learners can attenuate negative affect and remain behaviorally engaged. In parallel, learners may engage in trust recovery by recalibrating their confidence in the AI system’s usefulness or reliability despite its shortcomings. Within the proposed framework, cognitive reappraisal and trust recovery operate as complementary recovery mechanisms that mediate the relationship between expectation violation magnitude and sustained engagement. While CAT has traditionally been applied to academic stress and interpersonal challenges, extending it to AI-related disappointment foregrounds recovery as an active psychological process rather than an assumed outcome.

Yet the capacity to reappraise failures and rebuild trust does not occur in a vacuum. Digital divide and resilience theories highlight how structural conditions and personal resources shape learners’ ability to recover from AI disappointment. In China’s rapidly expanding AI-supported EFL ecosystem, disparities in internet connectivity, device access, and availability of alternative learning support remain substantial. According to the China Internet Network Information Center (2023), nearly 30% of rural learners lack reliable internet access, increasing their reliance on a single AI system as a primary tutor. Under such conditions, AI failures carry heightened psychological and opportunity costs, making disengagement more likely.

Within this context, digital grit—defined as perseverance in the face of digital setbacks—emerges as a critical resilience resource. Learners with higher digital grit are more likely to persist through expectation violations, remain engaged long enough for cognitive reappraisal to take effect, and allow trust recovery processes to unfold. Conversely, learners with limited grit or constrained access may disengage before recovery mechanisms can operate. Thus, digital grit conditions the strength of the relationships linking expectations, violations, recovery processes, and engagement, explaining heterogeneity in learner responses that cannot be captured by EVT or CAT alone.

**Figure 1:**
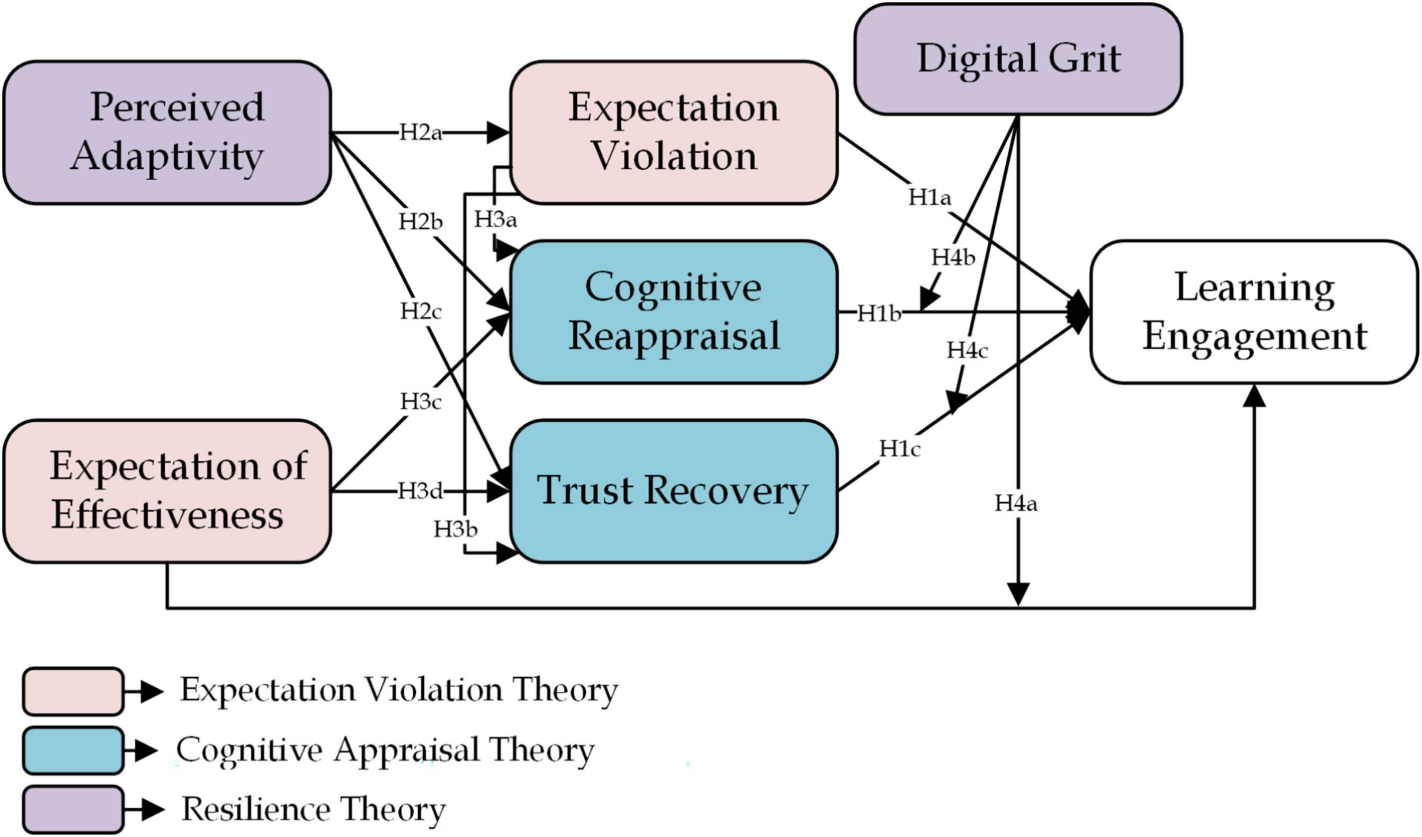
Research framework.

Taken together, this integrated framework specifies a coherent theoretical logic: expectation violation magnitude triggers engagement disruption, cognitive reappraisal and trust recovery function as mediating recovery mechanisms, and digital resilience shapes whether these mechanisms can operate effectively under varying contextual conditions. By embedding EVT, CAT, and digital divide/resilience perspectives within a single narrative, the model captures the full cycle of expectation formation, violation, appraisal, recovery, and persistence in AI-supported EFL learning. In doing so, it moves beyond adoption- and benefit-centric frameworks to offer a theoretically grounded explanation of when, how, and for whom AI supports sustained learner engagement (see Figure 1).

### 2.3 Hypothesis development

#### 2.3.1 Core violation–recovery pathways

Learners’ expectations are central to engagement, especially in digitally mediated education. EVT (Burgoon, 2015) posits that unmet expectations trigger cognitive and affective reactions. In AI-powered EFL learning, students expect adaptive feedback and contextual accuracy. When these are absent—such as an AI evaluator misclassifying grammar or producing repetitive comments—students experience expectation violation magnitude (EVM). Evidence shows unmet expectations undermine motivation and engagement. For example, course clarity and task relevance affect engagement through expectancy and task value beliefs (Vo and Ho, 2024), while social presence enhances expectancy and value, shaping behavioral and cognitive involvement (Edwards and Taasoobshirazi, 2022). Beyond education, expectation violations increase maladaptive coping and withdrawal when gaps between expectation and reality are large (Gesualdo and Pinquart, 2022). In AI-EFL contexts, repeated personalization failures may erode persistence, particularly where performance pressures are high.

*H1a*: Expectation violation magnitude negatively influences learning engagement.

Learners, however, are not passive recipients of disappointment. CAT (Lazarus, 1991) highlights the role of cognitive reappraisal—reframing negative events to sustain motivation. In education, reappraisal mediates the link between COVID-19 stressors and engagement (Buzdar and Ikram, 2024) and between teacher support and EFL learners’ engagement (Zhang et al., 2024). In AI-supported contexts, a student may interpret a chatbot’s error as a temporary glitch rather than systemic failure, maintaining participation. Yet reappraisal is not universally effective: Kremer et al. (2023) found it did not offset negative emotions in medical training, reducing study time and performance. Still, in resource-limited EFL environments, reappraisal may be vital to sustain engagement when AI is the sole tutor.

*H1b*: Cognitive reappraisal positively influences learning engagement.

A further recovery mechanism involves trust, central to educational relationships. Engagement strengthens when learners perceive systems as reliable and supportive (Payne et al., 2022; Bayraktar et al., 2025). Yet trust in AI is fragile; confidence erodes quickly when adaptive systems fail repeatedly. Research shows trust violations, though damaging, can be repaired through explanations and corrective action (Kanaris and Mujtaba, 2023). Psychological security derived from trust also predicts engagement and performance in online education (Tatiana et al., 2022). For example, if an AI platform improves over time—shifting from generic corrections to context-sensitive feedback—learners may rebuild confidence and re-engage. This mirrors findings in educational leadership, where trust-building fosters sustained engagement (Zhou et al., 2022). In rural China, where many students depend on a single AI tool, trust recovery is critical, as disengagement could severely hinder learning opportunities.

*H1c*: Trust recovery positively influences learning engagement.

#### 2.3.2 Role of perceived AI adaptivity

Perceived AI adaptivity—the ability of a system to tailor responses to learner needs—shapes expectation violations, emotional coping, and trust. EVT suggests that mismatches between expected and actual performance generate negative responses (Burgoon, 2015). Research shows users react more harshly to AI errors than to human ones, given heightened expectations of precision (Boo, 2024; Ryoo et al., 2025). In EFL contexts, an AI grammar checker that ignores context may elicit stronger disengagement than a human teacher’s mistake, as failures are perceived as a breakdown of the “machine advantage.” The adaptation gap—discrepancy between expected and perceived functions (Komatsu and Young, 2010)—is narrower when systems are adaptive, lowering violation magnitude.

*H2a*: Perceived AI adaptivity negatively influences expectation violation magnitude.

Adaptivity also enables cognitive reappraisal. CAT suggests that personalized cues help learners reframe disappointing experiences. AI-assisted interventions such as supportive visualizations reduce negative affect and build resilience (Pinzuti et al., 2025). In EFL education, adaptive tutors adjusting explanations to proficiency or offering culturally relevant examples allow learners to reinterpret errors as scaffolding rather than systemic flaws. By contrast, generic AI feedback limits reappraisal opportunities. Since reappraisal enhances persistence in adversity (Riepenhausen et al., 2022; Stover et al., 2024), adaptivity functions as both a technical affordance and a psychological enabler.

*H2b*: Perceived AI adaptivity positively influences cognitive reappraisal.

Finally, adaptivity strengthens trust recovery. Adaptive systems enhance perceptions of transparency and fairness, which build trust (Staab et al., 2025). In chatbot-based EFL learning, tailored responses promote trust through competence and social presence (Jiang et al., 2022). An AI tutor that recalibrates after errors—for example, moving from simple corrections to richer explanations—signals responsiveness, aiding trust repair. Research shows error timing matters: late-stage failures are more forgivable than early ones (Kahr et al., 2024), emphasizing the value of adaptive recalibration cues (Okamura and Yamada, 2020). In this way, adaptivity is not just functional but relational, helping learners renegotiate confidence in AI systems.

*H2c*: Perceived AI adaptivity positively influences trust recovery.

#### 2.3.3 Mediating recovery mechanisms: cognitive reappraisal and trust recovery

Expectation violations in AI-supported EFL learning do not necessarily lead to enduring disengagement because learners may activate recovery processes that regulate negative affect and restore willingness to continue. Consistent with Cognitive Appraisal Theory, recovery is shaped by how learners appraise the meaning of an adverse event and mobilize coping responses (Lazarus, 1991). In this study, two recovery mechanisms are emphasized: cognitive reappraisal and trust recovery. Cognitive reappraisal involves reframing an AI failure (e.g., inaccurate feedback) as temporary, situational, or system-limited rather than as a stable indicator of learner ability. Prior evidence suggests reappraisal can increase engagement and learning performance by regulating negative affect and sustaining effort, particularly when challenges are interpretable and controllable (Strain and D’Mello, 2014; Losenno et al., 2020; Datu et al., 2022; Buzdar and Ikram, 2024). At the same time, reappraisal is not universally effective; under high-stress or low-control conditions it may be insufficient to offset negative affect, which can reduce persistence (Kremer et al., 2023). These mixed findings imply that reappraisal is a plausible, but context-sensitive, pathway through which learners remain engaged after disappointment.

Trust recovery represents a complementary mechanism that operates at the relational level of learner–AI interaction. When AI violates expectations, learners may downgrade perceived reliability, competence, or benevolence of the system, thereby weakening willingness to rely on its feedback. Trust recovery reflects the extent to which learners rebuild confidence in the AI tool following a violation, a process widely shown to shape engagement and continued participation after service failures or perceived unfairness (Schilke et al., 2013; Honora et al., 2023; Mubashar et al., 2022). In learning contexts, trust is similarly central to sustained use of instructional technologies and to productive engagement in mediated environments (Poort et al., 2020; Schroeder et al., 2023). In AI-supported EFL tasks, trust recovery is particularly relevant because effective engagement often requires repeated reliance on AI feedback; without restored trust, learners may disengage even if they remain capable of effort.

Crucially, the relationship between cognitive reappraisal and trust recovery can be conceptualized in three ways. First, they may operate as independent and parallel mechanisms: learners can remain engaged by regulating their emotions through reappraisal even if trust remains low, or conversely may regain trust through system responsiveness even with minimal deliberate reappraisal. Second, they may operate sequentially, where reappraisal precedes trust recovery: by reframing the meaning of AI failure, learners stay engaged long enough to observe improvements, clarification cues, or adaptive responses that enable trust rebuilding (Lazarus, 1991; Schilke et al., 2013). Third, they may operate interactively, such that reappraisal is more effective when trust recovery is possible (or vice versa), implying that the combined presence of both processes produces the strongest engagement resilience.

Given the study’s focus on identifying core recovery channels and maintaining model parsimony, we examine cognitive reappraisal and trust recovery as parallel mediators linking expectation violations to learning engagement. This specification aligns with CAT’s view that multiple coping responses can be activated in response to the same negative event and can independently shape behavioral persistence (Lazarus, 1991). At the same time, we recognize that sequential or interactive recovery structures are plausible and should be tested in future research using longitudinal or experience-sampling designs that can capture temporal ordering and dynamic coupling between psychological coping and relational trust restoration.

Accordingly, the following hypotheses are proposed:

*H3a*: Cognitive reappraisal mediates the relationship between expectation violation magnitude and learning engagement.

*H3b*: Trust recovery mediates the relationship between expectation violation magnitude and learning engagement.

*H3c*: Cognitive reappraisal mediates the relationship between expectancy of AI effectiveness and learning engagement.

*H3d*: Trust recovery mediates the relationship between expectancy of AI effectiveness and learning engagement.

#### 2.3.4 Moderating role of digital grit

Although expectation violations, cognitive reappraisal, and trust recovery form the core pathways to learner engagement in AI-supported EFL contexts, learners differ substantially in their capacity to persist when AI systems underperform. Digital grit, defined as perseverance and sustained effort in technology-mediated learning environments, helps explain this heterogeneity. Extending Duckworth’s (2016) concept of grit to digital learning, digital grit captures learners’ willingness to continue engaging despite technology-specific obstacles such as unstable connectivity, opaque system logic, or repeated AI errors (Aparicio et al., 2017; Azari Noughabi and Ghasemi, 2024).

From an Expectation Violation Theory (EVT) perspective, digital grit buffers the negative effects of mismatches between expected and actual AI performance. EVT suggests that violations trigger negative affect and disengagement unless individuals tolerate or reinterpret the discrepancy (Burgoon, 2015). Learners with higher digital grit are more likely to endure such violations without immediately withdrawing, whereas low-grit learners tend to interpret unmet expectations as definitive failure, amplifying disengagement (Derakhshan and Fathi, 2023; Habib et al., 2025).

Digital grit also conditions the effectiveness of the recovery mechanisms proposed by Cognitive Appraisal Theory (CAT). Cognitive reappraisal requires sustained cognitive effort to reinterpret AI failure as temporary, system-limited, or non-diagnostic of personal ability (Lazarus, 1991). Similarly, trust recovery depends on continued interaction with the AI system over time to recalibrate expectations and perceived reliability. Empirical evidence in digital and language-learning contexts shows that grit facilitates persistence, emotional regulation, and adaptive coping, thereby strengthening the impact of reappraisal and trust repair on engagement (Resnik et al., 2021; Sulla et al., 2022).

The moderating role of digital grit is particularly salient in resource-constrained and rural contexts, where learners often rely on a single AI tool as a primary source of EFL support. Digital divide research indicates that limited connectivity, device access, and alternative instructional resources increase learners’ vulnerability to disengagement following technological failure (Warschauer, 2007; Van Dijk et al., 2016). In such settings, perseverance functions as an equity-relevant capability: learners with higher digital grit are more likely to sustain engagement despite AI shortcomings, whereas those with lower grit face compounded risks of disengagement and learning loss (China Internet Network Information Center, 2023).

Accordingly, digital grit is expected to moderate the relationships between expectation of AI effectiveness, cognitive reappraisal, trust recovery, and learner engagement:

*H4a*: Digital grit moderates the relationship between expectation of AI effectiveness and learner engagement.

*H4b*: Digital grit moderates the relationship between cognitive reappraisal and learner engagement.

*H4c*: Digital grit moderates the relationship between trust recovery and learner engagement.

## 3 Research methodology

### 3.1 Research design

This study employed a quantitative survey design with a two-wave time-lagged structure to reduce common method bias and strengthen temporal separation between predictors and outcomes (Podsakoff et al., 2012; Alam et al., 2025). A structured questionnaire was developed using validated scales and contextualized for AI-supported EFL learning environments. The research focused on widely used AI-assisted English learning tools in China, including grammar checkers, automated essay scorers, chatbots, and adaptive vocabulary tutors. These technologies were selected because they represent the most common interaction points where expectation violations and recovery mechanisms are likely to occur.

A three-week interval was employed between Wave 1 and Wave 2 for three reasons. First, this interval is sufficiently long to create meaningful psychological separation between measurements, thereby mitigating consistency artifacts and common method variance, while remaining short enough to minimize recall decay and sample attrition in student populations (Podsakoff et al., 2012; Dillman et al., 2014). Second, the study focuses on short-run cognitive–affective recovery processes following AI expectation violations rather than long-term developmental change; prior research suggests that appraisal and trust recalibration processes typically unfold within weeks rather than semesters (Lazarus, 1991; Schilke et al., 2013). Third, pilot feedback indicated that longer lags would substantially increase attrition among private institute learners with irregular attendance patterns.

No instructional intervention or experimental manipulation was introduced between the two waves. Participants continued their routine AI-assisted EFL learning activities, allowing the study to capture naturally occurring expectation violations and recovery responses. While longer lags (e.g., semester-based designs) may be valuable for examining longitudinal adaptation or skill development, they were not aligned with the present study’s theoretical focus on immediate recovery dynamics and would introduce confounds related to curriculum change, examination cycles, and instructor effects. This design choice is acknowledged as a boundary condition and addressed in the limitations section.

### 3.2 Sampling strategy and data collection

A stratified purposive sampling strategy was employed to ensure theoretically meaningful variation across socio-digital context and learning environment, consistent with the study’s equity-oriented objectives. Stratification was applied along two dimensions: geographic context (urban vs. rural) and educational setting (universities vs. private language institutes). This approach was chosen not to estimate population parameters, but to ensure sufficient representation of learner groups that are expected to experience AI expectation violations and recovery processes differently due to contextual constraints.

Guangzhou, a first-tier city and major EdTech innovation hub, represented the digitally privileged urban context, while surrounding towns and rural areas within Guangdong province represented resource-constrained settings. Guangdong was selected strategically because it combines advanced AI adoption with persistent digital divides, making it an appropriate setting to examine both privilege and constraint within a single regional system.

The final matched sample comprised 298 respondents across two survey waves. The distribution was 63% urban (*n* = 188) and 37% rural (*n* = 110); 69% university students (*n* = 205) and 31% private institute learners (*n* = 93). In Wave 1 (*N* = 321), data were collected on perceived AI adaptivity, expectancy of AI effectiveness, digital grit, and demographic variables. Wave 2, conducted 3 weeks later, yielded 298 matched responses (attrition rate = 7.2%) and captured expectation violation magnitude, cognitive reappraisal, trust recovery, and learning engagement. This attrition rate is well within acceptable thresholds for multi-wave survey research (Dillman et al., 2014).

Recruitment was conducted through multiple channels to reduce coverage bias. University students were recruited via institutional mailing lists, official WeChat course groups, and classroom announcements, with surveys hosted on Wenjuanxing (Questionnaire Star). Private institute learners were recruited through collaboration with two large English training centers, where QR codes linking to the survey were displayed in classrooms and reception areas. Eligibility required participants to have used at least one AI-assisted English learning tool for a minimum of 1 month. Participation was voluntary and anonymous, with no monetary incentives offered; instead, participants could opt to receive a summary of aggregated findings.

### 3.3 Measurement of constructs

All constructs were measured using established multi-item scales, carefully adapted to the AI–EFL learning context through translation, back-translation, and expert review. Responses were recorded on a 5-point Likert scale (1 = strongly disagree; 5 = strongly agree), which is widely used and culturally appropriate for Chinese survey research.

Perceived AI adaptivity was measured using items adapted from AI pedagogy and human–computer interaction research (Samuel et al., 2022; Zhao et al., 2022), capturing personalization, responsiveness, and contextual fit. Expectation violation magnitude (EVM) was modeled as a formative construct, grounded in Expectation Violation Theory, capturing discrepancies in perceived accuracy, adaptivity, and feedback relevance (Gesualdo and Pinquart, 2022). Cognitive reappraisal was measured using items adapted from the Emotion Regulation Questionnaire (Gross and John, 2003), contextualized for AI-assisted learning (Buzdar and Ikram, 2024; Zhang et al., 2024). Trust recovery was assessed using items adapted from technology trust literature, focusing on willingness to re-engage with AI tools following perceived failures (McKnight et al., 2002; Kanaris and Mujtaba, 2023).

Learning engagement was conceptualized as a higher-order reflective construct, comprising cognitive, behavioral, and emotional dimensions, operationalized using established engagement scales (Fredricks et al., 2004) validated in EFL contexts (Zhang et al., 2024). Digital grit was measured using items adapted from Duckworth et al.’s (2007) grit scale and refined using digital resilience research (Derakhshan and Fathi, 2023).

The questionnaire was originally developed in English, translated into Chinese, and back-translated following Brislin’s (1980) procedure. Content and face validity were reviewed by three EFL instructors and two AI education specialists. A pilot study with 50 students assessed item clarity, completion time, and preliminary reliability; minor wording refinements were made, and all Cronbach’s alpha values exceeded 0.70. Data analysis was conducted using PLS-SEM (SmartPLS 4) due to its suitability for formative–reflective models, complex mediation–moderation structures, and prediction-oriented research designs.

### 3.4 Mitigation of bias

Multiple procedures were implemented to reduce potential biases. Selection bias was mitigated through stratified purposive sampling that ensured representation across socio-digital contexts. Attrition bias was minimized by maintaining a short time lag and issuing structured reminders via WeChat and institute staff, resulting in a low attrition rate (7.2%). Social desirability bias was reduced by ensuring anonymity and emphasizing that there were no correct or incorrect responses (Podsakoff et al., 2012). To reduce coverage bias, data collection combined online surveys with in-person QR-code access, enabling participation from learners with limited internet access.

### 3.5 Ethical considerations

This study was conducted in accordance with the ethical principles of the Declaration of Helsinki and received ethical approval from the Human Resource Ethics Committee of Nanfang College, Guangzhou, China (Reference No: NCG20250312). All participants provided informed consent prior to participation. For online surveys, consent was obtained via a mandatory consent confirmation screen before questionnaire access; for QR-code-based in-person recruitment, a written consent statement was included at the beginning of the survey. Participation was voluntary, responses were anonymous, and participants were informed of their right to withdraw at any time without penalty.

## 4 Data analysis

The demographic distribution of the 298 valid respondents is presented in Table 1. A majority were female (59.4%, *n* = 177), with males accounting for 40.6% (*n* = 121). This pattern reflects the well-documented gender imbalance in English language learning in China, where female learners consistently outnumber males in both university and private institute contexts. Age distribution aligned with the target population of young adult EFL learners: 46.3% were aged 21–23 years, 27.5% were 18–20 years, 17.8% were 24–26 years, and only 8.4% were 27 years and above. This skew toward younger cohorts is consistent with the profile of students most actively engaged in AI-assisted English learning.

**Table 1:** Demographic of the respondents.

Variable	Category	*n*	%
Gender	Male	121	40.6
	Female	177	59.4
Age group	18–20 years	82	27.5
	21–23 years	138	46.3
	24–26 years	53	17.8
	27 years and above	25	8.4
Educational setting	University students	205	68.8
	Private institute learners	93	31.2
Region	Urban (Guangzhou city)	188	63.1
	Rural (Guangdong towns/villages)	110	36.9
Internet access	Stable broadband + mobile data	202	67.8
	Mobile data only	74	24.8
	Intermittent/low connectivity	22	7.4
Device ownership	Smartphone only	91	30.5
	Smartphone + Laptop/PC	163	54.7
	Shared device access	44	14.8
Experience with AI Tools	1–3 months	97	32.6
	4–6 months	126	42.3
	More than 6 months	75	25.1

In terms of educational setting, 68.8% were university students (*n* = 205), while 31.2% were private institute learners (*n* = 93), reflecting the stratified design. Regional distribution captured the urban–rural divide in Guangdong: 63.1% (*n* = 188) were drawn from Guangzhou city, one of China’s most digitally advanced urban centers, while 36.9% (*n* = 110) were from smaller towns and rural areas in Guangdong province.

Access to technology revealed persistent equity gaps. While two-thirds (67.8%) reported stable broadband plus mobile data, nearly a quarter (24.8%) relied solely on mobile data and 7.4% had intermittent connectivity. Device ownership further highlighted digital inequality: although 54.7% had both a smartphone and laptop/PC, 30.5% relied exclusively on smartphones and 14.8% depended on shared devices.

Finally, all respondents met the eligibility requirement of prior AI-assisted learning experience. About 42.3% had used AI tools for 4–6 months, 32.6% for 1–3 months, and 25.1% for more than 6 months. This distribution confirms that most participants had sustained engagement beyond the novelty phase, allowing robust evaluation of expectation violations and recovery mechanisms.

### 4.1 Measurement model

**Figure 2:**
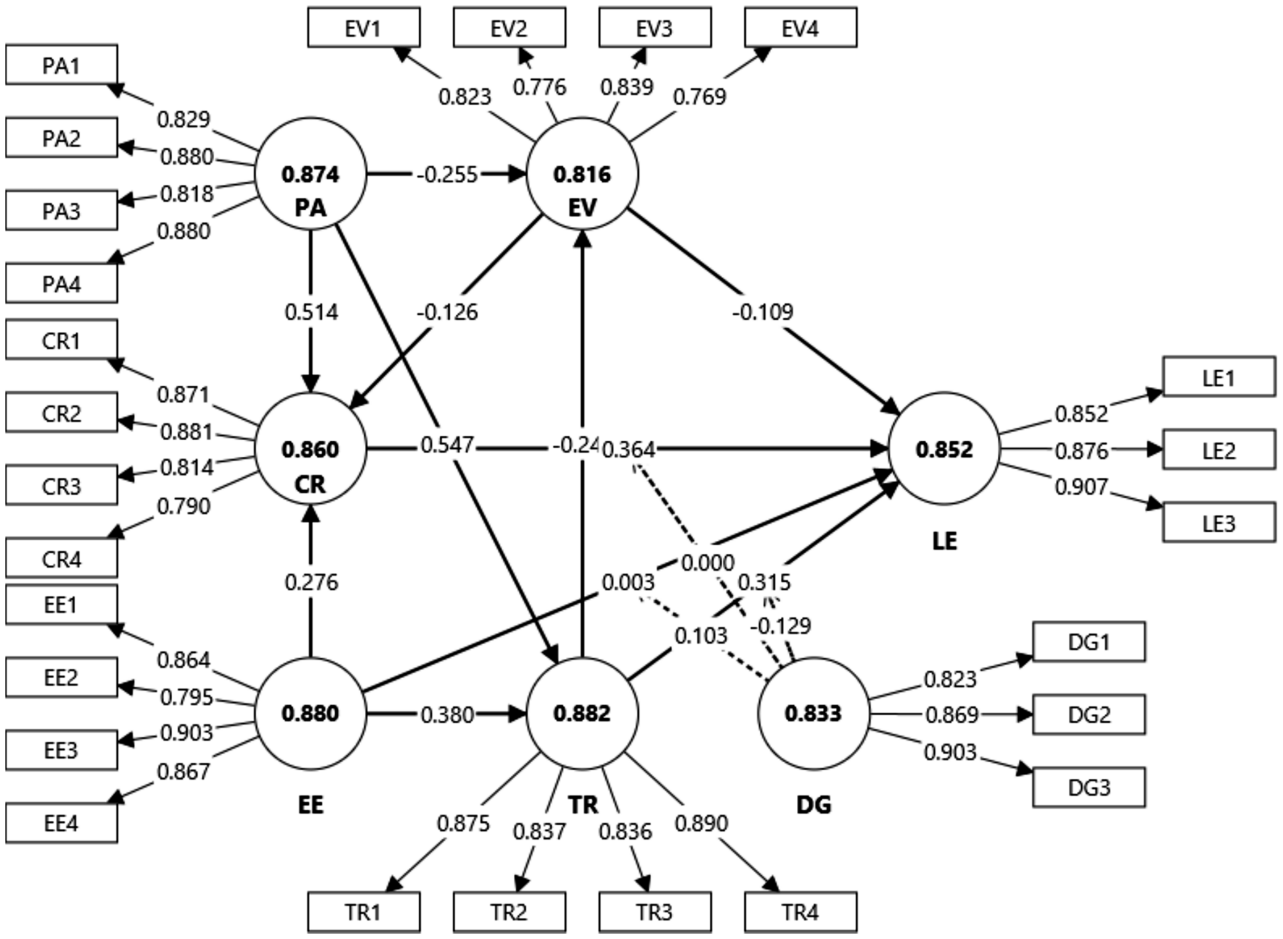
Measurement model.

Before testing the structural relationships, the quality of the measurement model was evaluated to ensure that all constructs demonstrated adequate reliability and validity. This assessment followed established criteria for Partial Least Squares Structural Equation Modeling (PLS-SEM), focusing on indicator reliability, internal consistency, convergent validity, and discriminant validity (Henseler et al., 2015; Hair et al., 2022) (see Figure 2).

#### 4.1.1 Indicator reliability and multicollinearity

Table 2 presents the results for outer loadings (OL) and variance inflation factors (VIFs). All items loaded strongly on their respective constructs, with values ranging from 0.766 (EV2) to 0.907 (LE3), comfortably above the recommended threshold of 0.70 (Alam et al., 2025). This indicates that each indicator contributed meaningfully to its latent construct. Although a few loadings were in the lower acceptable range (e.g., EV2 at 0.766), they were retained because their removal did not significantly increase composite reliability (CR) or average variance extracted (AVE), and they remained theoretically essential for capturing the construct domain.

**Table 2:** Measurement model statistics.

Variables	Items	OL	VIF	CR	AVE
CR	CR1	0.872	2.303	0.905	0.705
	CR2	0.880	2.428		
	CR3	0.814	1.964		
	CR4	0.790	1.757		
DG	DG1	0.823	1.808	0.900	0.750
	DG2	0.869	1.919		
	DG3	0.903	2.446		
EE	EE1	0.864	2.385	0.918	0.737
	EE2	0.795	1.760		
	EE3	0.903	3.047		
	EE4	0.867	2.796		
EV	EV1	0.827	1.796	0.878	0.643
	EV2	0.766	1.663		
	EV3	0.842	1.810		
	EV4	0.769	1.557		
LE	LE1	0.852	1.967	0.910	0.772
	LE2	0.876	2.049		
	LE3	0.907	2.493		
	PA1	0.829	2.159		
PA	PA2	0.880	2.647	0.914	0.726
	PA3	0.818	1.992		
	PA4	0.880	2.545		
TR	TR1	0.874	2.519	0.919	0.739
	TR2	0.836	2.010		
	TR3	0.837	2.108		
	TR4	0.891	2.773		

Multicollinearity was assessed using VIF values. All items recorded VIFs between 1.55 and 3.05, well below the conservative cut-off of 5 (Hair et al., 2022). This confirms that multicollinearity was not a threat to the model estimation, and each indicator provided unique variance to its construct.

#### 4.1.2 Internal consistency reliability

Internal consistency reliability was examined using both Cronbach’s alpha and composite reliability (CR). Since PLS-SEM prioritizes CR over Cronbach’s alpha, the focus was on CR values. As shown in Table 2, CR values ranged from 0.878 (EV) to 0.919 (TR), all exceeding the recommended threshold of 0.70 and approaching or surpassing 0.90, which indicates excellent reliability without suggesting redundancy (Nunnally and Bernstein, 1967). This demonstrates that the items within each construct consistently measured the same latent concept.

#### 4.1.3 Convergent validity

Convergent validity was assessed through the average variance extracted (AVE). All constructs achieved AVE values above the threshold of 0.50, ranging from 0.643 (EV) to 0.772 (LE), which indicates that each construct explained more than half of the variance in its items (Fornell and Larcker, 1981). This provides strong evidence of convergent validity. Importantly, the higher AVE values for constructs such as Learning Engagement (0.772) and Digital Grit (0.750) suggest that these constructs were measured with particularly strong precision and representativeness.

#### 4.1.4 Discriminant validity

Discriminant validity was assessed using both the heterotrait–monotrait ratio of correlations (HTMT) and the Fornell–Larcker criterion. Table 3 reports the HTMT results, which were all below the conservative threshold of 0.85 (Alam et al., 2025), with the highest observed value being 0.872 (PA–TR). This provides strong evidence that the constructs are empirically distinct from one another.

**Table 3:** Discriminant validity.

Variables	CR	DG	EE	EV	LE	PA	TR
HTMT							
CR							
DG	0.468						
EE	0.721	0.379					
EV	0.559	0.213	0.500				
LE	0.817	0.427	0.627	0.508			
PA	0.840	0.379	0.662	0.520	0.791		
TR	0.854	0.416	0.790	0.519	0.783	0.872	
FLC							
CR	0.840						
DG	0.397	0.866					
EE	0.629	0.324	0.858				
EV	−0.473	−0.174	−0.426	0.802			
LE	0.703	0.363	0.547	−0.432	0.878		
PA	0.731	0.323	0.583	−0.446	0.684	0.852	
TR	0.745	0.359	0.698	−0.443	0.681	0.768	0.860

The Fornell–Larcker criterion further confirmed discriminant validity, as the square roots of AVE (diagonal values) were consistently higher than the inter-construct correlations (off-diagonal values). For example, the square root of AVE for Learning Engagement was 0.878, which exceeded its correlations with Perceived AI Adaptivity (0.684) and Trust Recovery (0.681). This indicates that each construct shared more variance with its own indicators than with other constructs in the model, supporting conceptual distinctiveness.

Taken together, the measurement model demonstrates robust reliability and validity across all criteria. The strong indicator loadings confirm that the survey items were appropriate and meaningful for their constructs. The CR and AVE values indicate that the constructs were measured with consistency and precision, while the HTMT and Fornell–Larcker results provide assurance that the constructs are empirically distinct. These results establish a solid foundation for testing the hypothesized structural relationships.

### 4.2 Predictive relevance

The model’s predictive capability was evaluated using PLS-Predict with 10-fold cross-validation (Shmueli et al., 2019). As shown in Table 4, all Q^2^ predict values were positive (0.343–0.408), confirming predictive relevance (Hair et al., 2022). These values suggest that the model explains a meaningful share of variance in learning engagement, with magnitudes approaching the “medium” predictive power threshold.

**Table 4:** Predictive statistics.

MV	*Q*^2^ predict	PLS-SEM_ RMSE	LM_ RMSE	IA_ RMSE
LE1	0.343	0.977	0.977	1.206
LE2	0.396	0.816	0.782	1.05
LE3	0.408	0.812	0.802	1.056

RMSE comparisons provide further nuance. For LE2 (0.816) and LE3 (0.812), the PLS-SEM model outperformed the naïve benchmark (IA = 1.050, 1.056) and was competitive with linear regression (LM = 0.782, 0.802), indicating strong predictive accuracy. For LE1 (0.977), PLS-SEM matched the linear benchmark and still improved on the naïve model (1.206), reflecting robustness even on the weaker item.

Overall, the results demonstrate that the structural model yields moderate and practically meaningful predictive power for learning engagement. This provides empirical support that constructs such as expectation violation, cognitive reappraisal, trust recovery, and digital grit not only hold theoretical significance but also enhance out-of-sample prediction in AI-supported EFL contexts.

### 4.3 Hypothesis testing and discussion

**Figure 3:**
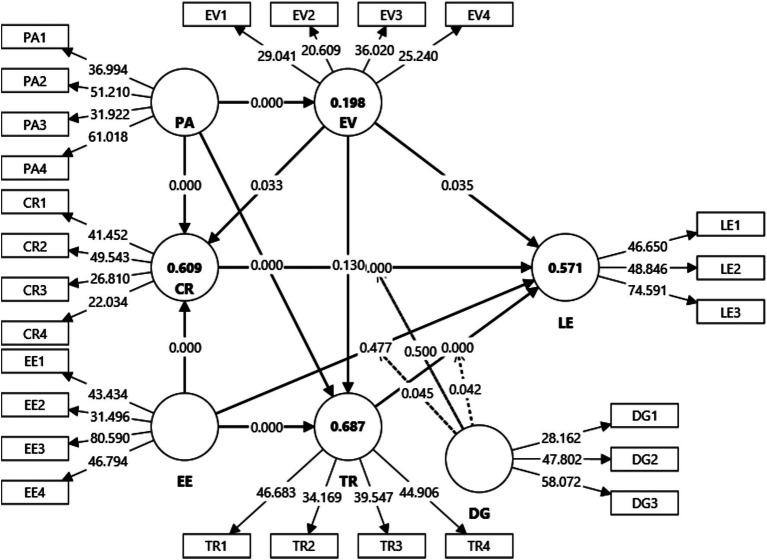
Structural model.

The hypothesized structural relationships were tested using bootstrapping with 10,000 resamples. Table 5 and Figure 3 summarize the standardized path coefficients, significance levels, effect sizes, and predictive confidence intervals. Overall, the model explains substantial variance in the key endogenous constructs (*R*^2^ = 0.571 for learning engagement; *R*^2^ = 0.609 for cognitive reappraisal; *R*^2^ = 0.687 for trust recovery; *R*^2^ = 0.198 for expectation violation magnitude), indicating strong explanatory power for a recovery-centered model in an emerging AI-supported EFL domain.

**Table 5:** Structural model statistics.

Hypothesis path		Std. Beta	Std. Div	*t* values	*p* values	*r*^2^	*f*^2^	PCI LL	PCI UL
H1a	EV - > LE	−0.109	0.060	1.810	0.035	0.571	0.019	−0.208	−0.014
H1b	CR - > LE	0.365	0.080	4.535	0.000		0.113	0.238	0.504
H1c	TR - > LE	0.315	0.084	3.730	0.000		0.080	0.183	0.456
H2a	PA - > CR	0.514	0.068	7.580	0.000	0.609	0.414	0.399	0.617
H2b	EV - > TR	−0.050	0.044	1.125	0.130	0.687	0.006	−0.124	0.020
H2c	PA - > EV	−0.445	0.067	6.625	0.000	0.198	0.247	−0.546	−0.323
H3a	EV - > CR - > LE	−0.046	0.025	1.828	0.034			−0.096	−0.013
H3b	EV - > TR - > LE	−0.016	0.015	1.080	0.140			−0.045	0.003
H3c	EE - > CR - > LE	0.101	0.028	3.568	0.000			0.063	0.158
H3d	EE - > TR - > LE	0.116	0.034	3.442	0.000			0.068	0.180
H4a	DG x EE - > LE	0.103	0.061	1.701	0.045		0.013	0.004	0.201
H4b	DG x CR - > LE	0.000	0.065	0.001	0.500		0.000	−0.111	0.101
H4c	DG x TR - > LE	−0.128	0.074	1.724	0.042		0.014	−0.248	−0.004

Addressing RQ1 (whether AI expectation violations undermine engagement), expectation violation magnitude (EVM) exerted a statistically significant but modest negative effect on learning engagement (H1a: *β* = −0.109, *p* < 0.05), consistent with Expectation Violation Theory (Burgoon, 2015) and prior evidence that unmet expectations reduce perceived value and persistence (Edwards and Taasoobshirazi, 2022; Vo and Ho, 2024). Importantly, the modest magnitude is theoretically informative rather than trivial: it suggests that violations matter, but they do not uniformly “collapse” engagement. This finding challenges a common implicit assumption in parts of the AI-in-education literature—namely that AI failure translates directly into disengagement—by showing that the direct damage is limited once recovery processes are accounted for. In other words, the primary story is not “AI failure → disengagement,” but “AI failure → recovery-dependent engagement.” That shift is exactly what success-biased adoption narratives typically overlook.

Turning to RQ2 (how learners recover), cognitive reappraisal emerged as the strongest predictor of engagement (H1b: *β* = 0.365, *p* < 0.001), supporting Cognitive Appraisal Theory (Lazarus, 1991) and corroborating evidence that reappraisal sustains engagement and performance under strain by regulating negative affect and preserving effort (Strain and D’Mello, 2014; Zhang et al., 2024). This result directly extends the current engagement literature in two ways. First, much work treats engagement as primarily driven by system features (usefulness, enjoyment, interactivity) or stable learner traits; by contrast, the current finding shows that engagement is strongly shaped by an event-based coping mechanism activated in response to disappointment. Second, many AI-EFL studies implicitly treat the learner as a passive recipient of AI quality; our results instead position the learner as an active regulator whose psychological recovery can outweigh the direct negative impact of AI failure.

Trust recovery also had a significant positive effect on engagement (H1c: *β* = 0.315, *p* < 0.001), consistent with scholarship emphasizing trust as essential for sustained engagement in technology-mediated learning (Payne et al., 2022; Bayraktar et al., 2025). However, the present study also complicates the way trust is often treated in prior research. Much of the trust literature assumes that trust follows performance (if the system is wrong, trust drops; if it is right, trust rises). Here, trust recovery appears less like a simple reflection of correctness and more like a relational recalibration shaped by cues of responsiveness and continued interaction (Kanaris and Mujtaba, 2023). This interpretation becomes clearer once the adaptivity and mediation results are considered.

Addressing RQ3 (the role of perceived AI adaptivity), perceived AI adaptivity (PA) proved central to the recovery architecture. PA strongly predicted cognitive reappraisal (H2a: *β* = 0.514, *p* < 0.001) and significantly reduced expectation violation magnitude (H2c: *β* = −0.445, *p* < 0.001). These results converge with the argument that adaptive systems narrow the “adaptation gap” by improving perceived fit between system output and learner needs (Komatsu and Young, 2010), and they align with emerging work suggesting that context-sensitive cues can support learner resilience in AI-supported learning (Pinzuti et al., 2025). Yet the critical contribution lies in what this implies for the broader literature: many studies operationalize “good AI” primarily as higher accuracy, and then infer engagement benefits. The present findings suggest a more precise mechanism—visible responsiveness and fit—through which AI supports engagement, even in environments where errors remain. This reframes design priorities away from pure performance metrics toward recovery-enabled interaction design.

A particularly diagnostic result is that the direct path from expectation violations to trust recovery was non-significant (H2b: *β* = −0.050, *p* > 0.1). This finding is theoretically important because it pushes back against a simplistic service-recovery analogy often assumed in technology trust discussions: that a failure automatically triggers “repair behavior.” Instead, violations alone do not produce trust rebuilding. Trust recovery appears to require conditions that make repair plausible—most notably perceived adaptivity and responsiveness. Put differently, learners do not rebuild trust simply because a system failed; they rebuild trust when they can see how the system is responding or when they can recalibrate how to use it effectively. This is a direct critique of models that treat trust recovery as a near-automatic response to violation intensity.

The mediation results sharpen the answer to RQ2 by specifying which recovery pathway is actually activated by violations. Cognitive reappraisal significantly mediated the relationship between expectation violation and engagement (H3a: *β* = −0.046, *p* < 0.05), whereas the parallel mediation through trust recovery was non-significant (H3b: *β* = −0.016, *p* > 0.1). This asymmetric pattern is not a minor technical detail; it directly corrects an ambiguity in current literature. Many studies discuss “coping” and “trust” together without specifying which mechanism carries the causal weight after failure. Here, the results indicate that violations reduce engagement primarily through cognitive strain that must be managed internally, not through a violation-triggered trust repair process. In contrast, expectancy of AI effectiveness influenced engagement through both cognitive and relational routes: the indirect effects via cognitive reappraisal (H3c: *β* = 0.101, *p* < 0.001) and trust recovery (H3d: *β* = 0.116, *p* < 0.001) were both significant. This suggests a dual pathway in which positive expectations energize engagement by strengthening both learners’ appraisal orientation and their willingness to restore trust when needed. Together, these findings refine EVT–CAT integration: reappraisal is the primary “violation-to-engagement” bridge, whereas trust recovery is more central to expectancy-driven motivation and continued reliance.

Addressing RQ4 (heterogeneity and boundary conditions), the moderating results reveal meaningful variation. Digital grit strengthened the expectancy of effectiveness → engagement relationship (H4a: *β* = 0.103, *p* < 0.05), indicating that perseverance in digital environments helps translate positive expectations into sustained learning behavior. The moderation of the reappraisal → engagement link was non-significant (H4b: *β* ≈ 0, ns), implying that once learners engage in reappraisal, its effect on engagement is relatively robust across grit levels. This finding is theoretically useful because it limits overly broad claims often made in the grit literature—namely that grit strengthens “everything.” Instead, the present evidence suggests a more selective role: grit matters most when persistence and continued reliance are required for motivational beliefs to become behavior.

Digital grit significantly moderated the trust recovery → engagement relationship (H4c: *β* = −0.128, *p* < 0.05). The negative interaction indicates that the effect of trust recovery on engagement varies by grit; one plausible interpretation is that high-grit learners are less dependent on trust restoration cues to remain engaged (they persist even when trust remains imperfect), whereas low-grit learners rely more heavily on trust rebuilding to sustain engagement. This offers a sharper critique of existing work that treats trust as uniformly central: for some learners, engagement persists even without full trust restoration, likely because perseverance sustains interaction long enough to keep learning behavior stable.

To make the linkage to the research questions explicit (and to avoid any “implicit mapping” weakness), the findings answer the RQs as follows. For RQ1, expectation violation magnitude negatively predicts engagement (*β* = −0.109), confirming that violations carry measurable costs. For RQ2, recovery mechanisms are decisive: reappraisal and trust recovery both positively predict engagement (*β* = 0.365; *β* = 0.315), but only reappraisal significantly mediates the violation → engagement pathway (H3a significant; H3b non-significant), indicating that violation-driven disengagement is primarily buffered cognitively. For RQ3, perceived AI adaptivity reduces violations and strongly enables reappraisal (PA → EVM negative; PA → CR positive), and violations do not directly drive trust recovery (EV → TR non-significant), implying that trust repair depends more on responsiveness cues than failure intensity. For RQ4, digital grit conditions whether expectancy and trust recovery translate into engagement (H4a and H4c significant), but does not alter the reappraisal–engagement slope, suggesting selective rather than universal moderation.

Finally, these findings reposition what it means for AI to “work” in AI-supported EFL learning. Much of the current literature implicitly equates effectiveness with accuracy and assumes engagement follows. The present results show that AI effectiveness must be evaluated in recovery terms: systems should be designed not only to minimize errors, but to enable learners to sustain engagement when errors occur, through adaptivity cues that activate reappraisal and make trust repair psychologically plausible. This recovery-centered account challenges adoption-focused and benefit-centric frameworks (e.g., simple usefulness → engagement logics) by demonstrating that AI learning is better understood as a cycle of expectation formation, violation, appraisal, and recovery—particularly in contexts where learners rely heavily on AI tools for language practice.

## 5 Implications of this study

### 5.1 Theoretical implications

This study advances theory by reframing Expectation Violation Theory (EVT), Cognitive Appraisal Theory (CAT), and Digital Divide/Resilience Theory in ways that move beyond their traditional applications. Collectively, the findings suggest that AI-mediated language learning requires a shift from adoption- or performance-centric models toward a recovery-centered paradigm, where violation, reappraisal, and resilience processes are foregrounded.

First, the results extend Expectation Violation Theory (EVT) into the domain of human–AI interaction in education. EVT has historically examined interpersonal communication, assuming that violations of social or behavioral norms generate predictable cognitive–affective responses (Burgoon, 2015). In this study, EVT is recontextualized: expectation violations emerge not from interpersonal breaches but from system errors such as generic or inaccurate feedback. Importantly, the findings reveal that violations do not uniformly predict disengagement. Their impact is contingent on subsequent recovery mechanisms and perceptions of adaptivity, suggesting that EVT must be expanded to account for dynamic violation–recovery cycles rather than static violation effects. This introduces a new theoretical nuance: in digital education, the *meaning of a violation is not fixed at the moment it occurs but is reconstructed through learners’ appraisal and relational repair processes.*

Second, the study deepens Cognitive Appraisal Theory (CAT) by positioning cognitive reappraisal as the central mechanism that converts disappointment into persistence. CAT traditionally frames reappraisal as an individual coping strategy in response to stress (Lazarus, 1991). Here, however, reappraisal functions not merely as coping but as a pedagogical bridge between failure and engagement in AI-supported learning. This suggests a novel application of CAT: in technologically mediated education, cognitive reappraisal is not only reactive but constitutive of engagement itself. Moreover, the finding that trust recovery mediates expectancy–engagement pathways highlights how appraisal processes extend beyond emotion regulation into relational recalibration with AI systems, a domain previously underexplored in CAT research.

Third, the integration of Digital Divide and Resilience Theory provides a macro-level extension to these psychological frameworks. Prior studies have often treated resilience or grit as individual traits (Duckworth, 2016; Van Dijk et al., 2016). By embedding digital grit into the model, this study demonstrates that resilience in AI-supported education is not a generic disposition but a situated capability shaped by structural inequalities in connectivity, device access, and learning opportunities. High-grit learners persisted long enough to benefit from trust recalibration, while low-grit learners disengaged prematurely. This finding reframes resilience as a mediating layer between structural inequities and micro-level coping processes, showing how digital resilience can amplify or dampen the benefits of EVT and CAT mechanisms in unequal educational contexts.

Taken together, these contributions suggest that the intersection of EVT, CAT, and Digital Resilience Theory provides a richer account of learning in AI-mediated contexts than any single lens alone. EVT explains the triggering condition (expectation violations), CAT explains the cognitive–affective recovery process (reappraisal and trust rebuilding), and resilience theory situates these within macro-structural inequalities that condition whether recovery is possible. The novelty of this study lies in demonstrating that these theories converge to form a dynamic, multi-level model of violation–recovery–engagement, shifting theoretical discourse from adoption to resilience, from static violation to iterative recovery, and from individual cognition to structural equity.

### 5.2 Practical implications

The findings of this study yield actionable lessons for educators, EdTech developers, and policymakers striving to make AI-supported English learning more effective and equitable.

First, for EdTech developers, the results highlight that the success of AI tools cannot be judged solely by accuracy benchmarks or efficiency metrics. Learners’ engagement is shaped less by whether an AI tool always gets the answer right than by how it responds when it gets things wrong. This shifts design priorities from error prevention to error recovery design. For example, instead of offering generic error messages (“incorrect grammar”), systems could deploy adaptive explanations that acknowledge the learner’s effort, clarify the misunderstanding, and provide scaffolds for re-engagement. Features such as transparency cues, personalization after mistakes, and trust-building dialogues can turn moments of violation into opportunities for relational repair. Developers should also integrate cognitive reappraisal prompts—short nudges that encourage learners to reinterpret errors as growth opportunities—directly into feedback systems, making recovery part of the design rather than an incidental learner strategy.

Second, for educators, the study suggests that teaching with AI requires a shift in pedagogy. Rather than presenting AI as an infallible tutor, instructors should normalize failure and recovery as part of the learning process. By modeling reappraisal strategies (e.g., “let’s see this error as a different way to practice”), teachers can help students develop resilience when systems disappoint. In high-stakes EFL contexts such as China, where learners often equate AI accuracy with exam readiness, explicit training in AI literacy—understanding strengths, limitations, and how to recover from errors—will be essential. Teachers can position AI not as a replacement but as a co-learning partner, reinforcing the idea that recovery from failure is as valuable as error-free performance.

Third, for policymakers and institutions, the integration of digital grit into the model underscores the need for policies that address structural inequities in access and resilience-building. Rural students, who often experience fragile connectivity and limited device ownership, are at greater risk of disengagement when AI systems fail. Policymakers should prioritize investments in infrastructure (stable broadband, affordable devices) while also embedding digital resilience training into curricula, ensuring learners acquire the psychological tools to persist through digital obstacles. Institutional policies can further promote equity by requiring EdTech vendors to test tools in under-resourced environments before wide adoption, avoiding designs that only succeed under ideal urban conditions.

Finally, across all stakeholders, this study points to the importance of reframing AI-supported learning as a recovery-centered ecosystem. Instead of evaluating AI tools solely by their capacity to deliver adaptive content, the focus should extend to how systems, educators, and institutions collectively help learners re-engage after expectation violations. In practice, this means designing trust-sensitive AI systems, cultivating emotionally intelligent pedagogies, and implementing resilience-oriented policies that recognize both the promise and fragility of AI-enhanced EFL education.

## 6 Limitations and further studies

Several limitations should be considered when interpreting the findings of this study.

First, although a two-wave time-lagged design was used to mitigate common method bias, the data rely on self-reported measures. While subjective appraisals are central to EVT and CAT, future studies could enhance robustness by integrating objective behavioral data (e.g., system logs, clickstream data, time-on-task) to capture recovery dynamics more precisely.

Second, the study examined short-term recovery processes following AI expectation violations. Learners’ expectations, trust calibration, and resilience may evolve over longer periods. Longitudinal or experience-sampling designs could reveal how recovery unfolds across semesters, examination cycles, or sustained AI use.

Third, the sample was drawn from a single Chinese province, limiting generalizability. Cultural norms, instructional traditions, and digital infrastructure vary across regions and countries. Cross-regional and cross-national replications are needed to test the boundary conditions of the proposed recovery model.

Fourth, recovery was modeled primarily at the learner level. However, AI-supported learning occurs within broader pedagogical systems. Future research could incorporate teacher mediation, peer support, and institutional scaffolding to examine multi-level recovery processes.

Fifth, the study did not differentiate among types of AI tools (e.g., chatbots, grammar checkers, automated essay scoring). Expectation formation and violation may differ across task types and evaluative stakes. Tool-specific or task-specific studies would refine theoretical precision.

Sixth, digital grit was treated as a relatively stable disposition. Yet resilience may be contextually cultivated through AI literacy training, instructional framing, or system-level scaffolds. Experimental or intervention-based research could test whether resilience can be actively developed, particularly among digitally disadvantaged learners.

Finally, the model focused on performance-related failures and did not address other emerging sources of expectation violation, such as algorithmic bias, opacity, or privacy concerns. Future studies should examine whether such violations activate similar or distinct recovery mechanisms.

Together, these limitations highlight the need for more longitudinal, multi-method, and multi-level research to deepen understanding of recovery-centered AI-supported learning.

## 7 Conclusion

This study advances AI-in-education research by shifting attention from adoption and performance to expectation violation and recovery. Integrating Expectation Violation Theory, Cognitive Appraisal Theory, and Digital Divide/Resilience Theory, the findings demonstrate that AI failures do not inevitably undermine learner engagement. Instead, engagement is sustained through cognitive reappraisal, trust recovery, and digital grit, particularly when AI systems signal adaptivity and responsiveness.

Using a two-wave survey of Chinese EFL learners, the study shows that expectation violations negatively affect engagement, but that this effect is substantially buffered by recovery mechanisms. Perceived AI adaptivity reduces violation magnitude and activates recovery, while digital grit conditions learners’ capacity to persist following AI failure. These results reconceptualize AI not merely as a personalization tool, but as a learning environment in which disappointment, emotional regulation, and resilience are central to sustained engagement.

Theoretically, the study extends EVT to human–AI educational interaction, reframes CAT as a mechanism of engagement recovery rather than mere coping, and situates resilience within socio-digital inequality. Practically, it underscores the importance of recovery-aware AI design, pedagogies that normalize AI imperfection, and policies that address digital disadvantage alongside technological innovation.

In conclusion, the effectiveness of AI in EFL education depends not only on system accuracy, but on how well learners and institutions recover when AI falls short. By foregrounding recovery and resilience, this study provides a foundation for more emotionally responsive, equitable, and sustainable AI-enhanced language learning environments.

## Data Availability

The raw data supporting the conclusions of this article will be made available by the authors, without undue reservation.

## Appendix: measurement items

**Table:**

Construct	Item code	Revised measurement item (single-barrel)	Source
Cognitive Reappraisal (CR)	CR1	When the AI tool gives feedback that disappoints me, I try to view the situation in a more positive way.	Gross and John (2003), Buzdar and Ikram (2024), and Zhang et al. (2024)
	CR2	I reinterpret AI mistakes as part of the learning process rather than as personal failure.	
	CR3	When AI feedback frustrates me, I try to change how I think about the situation.	
	CR4	I remind myself that AI errors do not reflect my true English ability.	
Digital Grit (DG)	DG1	I continue using AI learning tools even when they do not work as expected.	Duckworth et al. (2007) and Derakhshan and Fathi (2023)
	DG2	I persist with AI-assisted English learning despite encountering problems with the system.	
	DG3	I keep trying to learn English with AI tools even when the learning process becomes difficult.	
Expectancy of AI Effectiveness (EE)	EE1	Before using the AI tool, I expected it to provide accurate support for English learning.	Expectancy–confirmation logic
	EE2	I believed the AI tool would improve my English learning efficiency.	
	EE3	I expected the AI tool to adapt to my learning needs.	
	EE4	Overall, I had high expectations for the effectiveness of the AI tool in my English learning.	
Expectation Violation Magnitude (EV)	EV1	The AI tool’s feedback was less accurate than I expected.	Gesualdo and Pinquart (2022)
	EV2	The AI tool adapted to my learning needs less than I expected.	
	EV3	The AI tool’s feedback was less relevant to my learning tasks than I expected.	
	EV4	Overall, the AI tool performed worse than I expected.	
Learning Engagement (LE)	LE1	I concentrate deeply when using AI tools for English learning.	Fredricks et al. (2004) and Zhang et al. (2024)
	LE2	I actively participate in AI-supported English learning activities.	
	LE3	I feel interested when learning English with AI tools.	
Perceived AI Adaptivity (PA)	PA1	The AI tool adjusts its feedback based on my English proficiency level.	Samuel et al. (2022) and Zhao et al. (2022)
	PA2	The AI tool responds differently depending on my learning needs.	
	PA3	The AI tool provides feedback that fits my learning context.	
	PA4	Overall, the AI tool behaves in an adaptive manner.	
Trust Recovery (TR)	TR1	Even after the AI tool makes mistakes, I am willing to continue using it.	McKnight et al. (2002) and Kanaris and Mujtaba (2023)
	TR2	I am still willing to rely on the AI tool after it makes errors.	
	TR3	My confidence in the AI tool can be restored after problems occur.	
	TR4	I am willing to give the AI tool another chance after it disappoints me.	
